# Involuntary and Persistent Environmental Noise Influences Health and Hearing in Beirut, Lebanon

**DOI:** 10.1155/2012/235618

**Published:** 2011-10-13

**Authors:** Marjaneh M. Fooladi

**Affiliations:** ^1^Council for International Exchange of Scholars, A Division of the Institute of International Education, 3007 Tilden Street NW, Suite 5-L, Washington, DC 20008, USA; ^2^College of Nursing, Florida State University, Vivian M. Duxbury Hall, 98 Varsity Way, P.O. Box 3064310, Tallahassee, FL 32306-4310, USA; ^3^School of Nursing, American University of Beirut, P.O. Box 11-0236, Riad El Solh, Beirut 1107 2020, Lebanon

## Abstract

*Objective*. This study was conducted to assess the effects of involuntary and persistent noise exposure on health and hearing among Lebanese adults in Beirut, Lebanon, where people are exposed to noise from construction sites, power generators, honking cars, and motorcycles. *Methods*. Using a descriptive and exploratory design with mixed methods, participants were surveyed, interviewed, and tested for hearing while street noise levels were measured near their residents and work places. *Results*. Self-reports of 83 Lebanese adult, who lived and worked in Beirut, helped identify common patterns in experiences such as irritability, anger, headaches, and sleep disturbances due to noise annoyance. Of those tested, 30% suffered from high-frequency hearing impairment. Our results showed that environmental sound dB had increased by 12% and sound intensity by 400% above the maximum standard level when compared to the WHO report of 1999. *Conclusion*. Environmental noise contributes to premature hearing loss and potentiates systemic diseases among Lebanese.

## 1. Introduction

The unrecognized effects of persistent exposure to environmental noise on health and hearing and the unexplored nonspecific responses to noise pollution in Beirut, Lebanon, were the focus of this study. Literature [1] reports have identified excessive external noise and smoking as a major cause of hearing loss and impairment among children and young adults. The nonmodifiable contributing risk factors are listed as age, genetic trends, male gender, and race. Modifiable factors are voluntary exposure to loud music, smoking, sports, diet, dental hygiene, and systemic diseases [1, 2].

The city of Beirut is a metropolitan and multicultural venue with high-rise buildings erected to replace traditional one-family dwelling and occupy a vast portion of the Mediterranean shores in condensed populated areas. The environmental noise associated with ongoing power generators and construction sites begins at dawn and continues through the night as honking cars and motorcycles zigzag through crowded narrow streets. There is no escape from noise since commercial and residential zones are adjacent in close proximity where people live above the shops and businesses. Incessant honking is an expression of frustration when narrow streets are blocked by parked cars on both sides and fruit venders at each intersection. 

In a surveyed community response to noise in Beirut, Lebanon [2], participants identified the main sources as motorcycles (70.4%), traffic (63.1%), and car horns (56.3%). Reported construction and generator annoyance were 42% and 55.1%, respectively. This study aims to identify the current level of persistent environmental noise effect on hearing and overall health among Lebanese living and working on Hamra in Beirut, Lebanon. 

In a study of 440 local residents of Beirut, ages 21–50, researchers examined the effects of environmental noise and smoking on hearing loss [2]. They divided the participants into 4 groups of smokers and nonsmokers living in noisy (70–90 dBA) and quiet areas (45–55 dBA) and found hearing loss among the smokers when exposed to 8000 Hz at high frequencies and more hearing impairment among smokers above age 40^+^. Younger nonsmokers (21–39) showed significant hearing impairment at low frequencies. 

Environmental noise and health were found [3] closely related when researchers assesses 105 adults living near Auckland International Airport in New Zealand for noise sensitivity and annoyance for any adverse health effects. They identified that noise contributed to sleep disturbance and reduced health-related quality of life. Similarly, 2,312 people living near Frankfurt Airport [4] were given environmental (EQoL) and health-related (HQoL) quality of life instruments to assess their experiences with aircraft noise annoyance and disturbances to their life quality. Researchers compared their findings from aircraft noise with exposure to road traffic and railway noise, and results suggested a recursive relationship between noise and health. 

Numerous empirical studies have identified long-term exposure to noise as a major health concern. Noise from siren or ambulance can generate an immediate protective reaction known as fight or flight. In a substantial review [5] traffic noise effects were found as a source of environmental annoyance. Referencing the Environmental Expert Council (EEC) of Germany, noise was reported as a major source of severe annoyance and distress. The fastest and most urgent signal to noise is mediated by a subcortical area on amygdala. Even during sleep, the environmental noise from airplanes or heavy equipments can generate dangerous brain signals to release stress hormones. The chronic release of stress hormone from long-term exposure to environmental noise increases the endogenous risk factors such as ischemic heart disease and myocardial infarction [5]. Because each individual study on the adverse health effects of noise in most cases does not reach statistical significance, meta-analysis of multiple studies [5] according to EEC shows a consistent trend towards cardiovascular risk when daytime noise level exceeds 65 dB(A). However, the urgent call for public health safety to reduce extra-aural occupational noise remains misclassified and warnings ignored. 

Unprotected and regular exposure to occupational loud noise in the daytime interferes with nocturnal sleep patterns. Researchers [6] divided 3 groups of 8 subjects (*n* = 24) and exposed them to continuous noise at >75 dB for 1–2 years, 5–10 years, and over 15 years and matched them with corresponding healthy control groups who worked in a quiet environment. After using PolySomnoGraphy (PSG) for an all-night sleep study, subjects rated their sleep quality on a Visual Analogue Scale (VAS), and the results showed that workers who were exposed to occupational noise had poor quality sleep and over the years adapted to noise. However, the long-term adverse health effects of noise hidden in an adaptive process or subsequent systemic diseases were not investigated. Assessing the sleep quality of air travelers and their exhaustion level after sleeping on flights may better reveal brain responses to unrecognized and constant noise. 

According to the 1998 WHO report [7], Lebanon occupies 10, 452 square kilometers (Km^2^) of land strip with a population of 4.5 million residents. Over 80% of Lebanese reside in crowded urban areas in high-rise buildings. Researchers [8] have examined the quality of life in high-rise buildings or controlled built environments (CBE) and found them incompatible with the psychological needs of women and children. The recent social architects place multiple families in one high-rise building, and residents exposed to overcrowding, external loud noise from proximate airports or railroads, air pollution, toxins, and malodorous sewer are those experiencing nonspecific stress manifested as irritability and aggression similar to patients in hospitals and convalescent centers or air travelers kept in CBEs such as airports who experience stress. 

Farmers using farming equipment are at risk for noise-induced hearing loss [9]. Ninety three industrial farmers between the ages of 18–75 were surveyed after complete health history and demographic data on noise exposure. Their bilateral hearing sensitivity was assessed, using air conduction audiometric testing at 500–800 Hz frequencies. Farmers completed the self-assessment of communication (SAC) hearing handicap scale, and their hearing threshold was less than 25 dB HL. Subjects had high-frequency hearing loss, and those older than age 50 had a higher perceived hearing handicap and damage from external noise. Among other noise-related health effects researchers [10, 11] have identified impaired cognitive abilities as reflects in Table 1, when at various noise levels there is annoyance, speech and sleep interference, reduced work productivity, hearing impairment, and physiological changes [10–13]. 

Noise at a certain frequency and decibel is detrimental to hearing and according to the United States Environmental Protection Agency (USEPA) and World Health Organization (WHO) (see Table 2) outdoor exposure to the environmental noise should remain within an acceptable range to avoid hearing loss [10–13]. According to the WHO report published by the Lebanese Ministry of Environment, the noise level in Beirut, Lebanon could have adverse health effects on the local residents (see Table 3). The physiological effects of environmental noise have been associated with the endocrine changes [14, 15].

## 2. Methodology

The purpose of this study was to examine the local perceptions on environmental noise levels and how exposure to involuntary and persistent noise affected young Lebanese health and hearing. To assess street noise level and its health and hearing effects, a cross-sectional design was adopted using mixed methods and the critical theory of participatory action according to Freire [16]. This exploratory and descriptive investigation was focused on individual experiences with persistent noise. 

Assured of anonymity and confidentiality participants willingly engaged in informal dialogues and interviews for 30–60 minutes. Interviews took place at various locations including workplace, shops, dormitories, or outdoors. Interviewers helped participants recall and explain past and present experiences with noise at different peak hours and describe how noise influenced their lives and probed for any pattern changes in sleep quality, appetite, and unrecognized stress. This study was initiated in September 2009 and ended in May 2010. Written consent in English and Arabic emphasized no risk for voluntary participation. Basic human rights to freedom were closely observed, and participants were given an opportunity to withdraw from the study at any time.

### 2.1. Data collection

A multidisciplinary research team of nursing, medicine, and engineering with cultural literacy retrieved ethnographic data according to Spradley [17]. Standard demographic survey questionnaire obtained personal data on education, employment, income, and interviews using open-ended questions focused on past and present health and hearing status and personal approach to stress management. Verbal description and self-report offered valuable personal experiences and concurrently were compared with literature. Recorded and expanded data reached saturation upon two rounds of transcription and were analyzed. Further networking with participants provided sufficient opportunities for clarification. Data were safely stored for future reference.

The initial proposal was aimed to include 100 equally divided men and women, and after randomly approaching residents and shop keepers on Hamra Streets we could only recruit 83 volunteer adult Lebanese (46 men and 37 women). Researcher and two graduate assistants from nursing administered survey questionnaire and interviewed participants for personal experiences with noise. One research assistant from engineering measured street noise levels in areas where participants worked and lived. Research team worked in rotations and frequently compared data to match participants with locations for increased data accuracy. Informal and semistructured interviews using open-ended questions helped retrieve participants' experiences and perceptions with noise to identify their ways of coping or adjusting to environmental noise. Participants were offered a small incentive to compensate for their time and cooperation. Anonymity was assured, and raw data was secured in locked office cabinets. 

Demographic data provided age range, the number of years working and/or living on premises, income, and education level. Interviews helped obtain information on health habits such as diet, sleep, and coping techniques. Later, participants were tested for hearing using pure tone screening to determine hearing impairment or loss. The street noise levels were measured at 4 busy intersections near participant's home and workplace using sound level meter (SLM). 

Our inclusion criteria consisted of Lebanese men and women between the ages 18–38 who lived and worked on Hamra Street for longer than 6 months. We chose a younger age group to eliminate age-related hearing loss or impairment and health complications associated with advanced age. Because this study was conducted in a metropolitan area and the majority of people in Beirut are trilingual (Arabic, English, and French) we chose to include people with basic language literacy (read and write) and unexposed to voluntary noise such as loud music.

### 2.2. Findings/Results

Data were analyzed through an immediate debriefing after each interview and compared with field notes and visual clues. Data was reviewed line by line and coded using the four phases of qualitative study by Polit and Hungler [18]. Author's personal experiences with noise annoyance verified published literature to substantiate scientific adequacy and data credibility. Verified data were compiled upon saturation and analysis. The inquiry audit and neutrality were established for dependability and data conformity. Transferability of the framework was unanimous among scholarly colleagues and their personal reflections on noise annoyance. 

Researcher and two assistants randomly approached people at various shops, stores, apartment buildings, and dormitories on Hamra Street where residents and shopkeepers often gathered to visit. Consenting participants were later met at a location of their choosing to be surveyed and interviewed. On the same day an appointment was made for each participant to meet individually or as a group for hearing test at the audiology clinic. In three occasions when 4-5 participants were waiting for hearing test, focus group discussion was held ranging from 60–90 minutes to explore more experiences with noise. At other occasions interviews were held at the store, at home, in dormitory, or outdoors. 

Standard demographic data revealed an average age of 26.5 for men and 27.3 for women, with mixed educational, income, and employment backgrounds. At interviews participants were asked to elaborate on their daily experiences with environmental noise, and 70% (58) had difficulty sleeping at night, and according to the noise meter results they were exposed to Leq 12 hr >65 dBA. In support of our findings multiple studies [7, 19, 20] have reporting the degree of annoyance, sleep disturbance, and hearing loss related to traffic noise in residential urban communities. Using a questionnaire on the influence of environmental noise on health, researchers [19] sampled 1000 individuals ages 19–80 in a heavy traffic area in Stockholm and found frequent annoyance among 13% of subjects exposed to Leq 24 hr >50 dBA when compared to the 2% who were exposed to <50 dBA. Sleep disturbance were reported by 23% at Leq 24 hr >50 dBA and by 13% exposed to <50 dBA. Habituation to noise has been more related to sleep rather than annoyance. Annoyance and sleep problems were prevalent among those with bedroom windows facing the streets or living in apartments [21]. 

Most studies on noise report human responses, and few have explored coping with constant noise. Our study focused on finding various forms of coping skills when participants shared experiences with increased craving for sweets 55% (45), caffeine intake 62% (51), and smoking 78% (65). More women 81% (30) in the study reported frustration, anger, and feeling helpless as an aftereffect of persistent noise. Men 93% (43) revealed habituation to noise by unrecognized and later described techniques such as chewing gums or tooth picks, snacking at work, and managing to sleep at night by using other incessant noise such as bathroom fan, radio, and television. Afternoon headaches were common among women 78% (29). The unexpected fear of knowing about hearing status 96% (80) among men and women revealed that majority of participants had suspected some hearing impairment when family members complained about having to speak louder or repeat themselves. 

Literature support identifies imbalance in endocrine system linked to emotional disturbance. Researchers [21, 22] remain uncertain about the habituation to stress and posit that repeated exposure to external stimuli can shape adaptive brain plasticity mechanism in response to homotypic challenges when cortical auditory processing areas respond to repeated loud noise.

Study limitations were few including low budget funding, time line for project completion per 10 months Fulbright contract, long delays for obtaining IRB approval to proceed with data collection, and participants who were business owner and could not leave their work to be tested for hearing or feared results. 

Participants experienced the physical and emotional effects of persistent noise in form of irritability, anger, nausea, headache, and sleep disturbances. Men 93% (43) mainly reported nervous eating, chewing (gum or tooth picks), and both genders 78% (65) smoked to relieve agitating discomforts (described as auditory disturbance on an aircraft). Participants showed concerns for recent weight gain 51% (42), hypertension 46% (38), and diabetes 54% (45). We assessed participant's hearing, and our measurable indicators and output activities were achieved using audiometer pure tone testing. We found bilateral hearing sensitivity upon assessment by air-conduction audiometric testing at 500–4000 Hz frequencies. Of those tested 40% (33), there were 30% (10) who suffered from asymmetrical high-frequency hearing loss and impairment. 

Street noise levels were measured by sound level meter (SLM) near participant's home and workplace at 4 intersections on Hamra and Bliss streets. We selected similar time frames and durations to match the WHO study of 1999 for increased accuracy in comparison. Tabulated and analyzed data showed an increase in environmental noise level between 1999 and 2009. The mean average noise standard dBA by the WHO report during 7 am to 6 pm was 55–65, and in 2010 it was 65–75 with a 12% increase in sound dB and 400% increase in noise intensity (Tables 4 and 5).

## 3. Discussion

As discovered, Lebanese cope with persistent noise in various forms such as smoking, consuming strong coffee, and sweets. To describe caffeine effects on stress response associated with noise, researchers [22] have reported that high doses of caffeine, a psychoactive substance, can activate hypothalamus-pituitary-adrenal axis (HPA), and low doses of caffeine can restrict some of the stress responses by HPA axis. Also, elevated stress due to loud noise was assessed [23] to find how noise influenced adrenocorticotropic hormone (ACTH) and corticosterone levels in animals with and without caffeine intake. Plasma ACTH and corticosterone levels peaked 30 minutes after exposure to noise and rapidly declined when noise was eliminated. Then low-dose caffeine at 2 mg/kg was injected to find a significant increase in plasma corticosterone and ACTH levels within 30 minutes and return to baseline after 60 minutes. When higher doses of caffeine (30 mg/kg and higher) were injected, the hormone levels increased and lasted for at least 2 hours in response to loud noise. 

In this study participants experienced various noise-related physical responses. Researchers [6, 22, 23] showed perceived threat from loud noise on HPA axis and the release of ACTH as corticosterone. Exposure to noise at night in acoustic chambers using various intensities showed elevated plasma ACTH and corticosterone at 85 dBA. Many regions of the brain showed negative response to noise due to audiogenic stress which provoked HPA axis with life-threatening responses.

We found participants expressing rage and frustration by honking and shouting when they could not control noise exposure. Similarly, an investigation [24] of 10 healthy human volunteers who were exposed to loud noise at 100 dB under controllable and uncontrollable conditions on two separate days revealed noteworthy results. Participants self-rated their responses, and both groups reported a higher sense of helplessness, lack of control, tension, stress, unhappiness, anxiety, and depression. There was a greater HPA axis response higher plasma adrenocorticotropic hormone levels and increased levels of sympathetic nervous system electrodermal activity among those exposed to uncontrollable noise [22–24]. Therefore, losing control over aversive stimulus can negatively affect mood and overall health. 

This study with literature support highlights many aspects of noise influence on health. The long-term exposure to the environmental noise can pose a threat to health by triggering responses from neuroendocrine and autonomic nervous system.

## 4. Conclusion

In a nonspecific and unrecognized way, noise can generate an unsettling level of stress with profound influence on general health. Upon our initial approach, Lebanese seemed fully aware and yet resigned toward noise problem. Common thread for adaptation to noise in this study was reported as a series of unhealthy habits and public expression of anger and rage by shouting and honking. Noise and uninvited sounds [25] adversely influence physical and psychological health. Lebanese men and women exposed to chronic noise and tested showed hearing loss at high frequency range, and those who could not attend testing sessions due to long work hours expressed fear of hearing impairment according to family members' complaints for having to speak louder or repeat themselves. 

In the United States, nearly 10 million adults and 5.2 million children suffer from irreversible noise-related hearing impairment, and 30 millions are at risk for daily exposure to dangerous levels of noise. The noise-related health effects are often ignored and yet significant such as hypertension, tachycardia and elevated cortisol levels, and stress [9, 12, 24]. 

Finally, our results showed that environmental sound dB had increased by 12% and sound intensity by 400% above the maximum standard level when compared to the WHO report of 1999 which confirms noise as an increasingly recognized global problem with consequential effects on life quality. This study was an attempt to bring awareness to how noise could affect hearing and over time influence daily behavior leading to systemic diseases. Although this study included seemingly healthy young adults (ages 18–38), we still found them at risk for non-age-related hearing loss. Potential for temporary or permanent loss of hearing among children and young adults warrants greater focus on public education and awareness on noise hazards.

## Figures and Tables

**Table 1:** Physiological and psychological effects of noise pollution.

Effect	Comment
Annoyance	Even relatively low levels of noise can cause annoyance and frustration. A tranquil background can make noise more intrusive. Natural sounds are generally less annoying than unnecessary or controllable sound such as car horns. For instance, intermittent sounds such as a tap dripping on a quiet night can be more disturbing than the sound of falling rain.

Speech interference	Noise can interfere with speech. When the background noise level is 50 dBA, normal conversation can be easily carried with someone up to 1 m away. Any more than that, problems will arise.

Sleep interference	Noise can wake people from sleep and keep them awake. Even if not actually woken, a person's sleep pattern can be disturbed, resulting in a reduced feeling of well-being the next day. External noise measuring up to 30 dBA in a bedroom is appropriate for sleep.

Decreasedwork performance	Noise pollution can make people nervous. Accordingly, it can prevent people from concentrating on their work. As noise levels increase, ability to concentrate and work efficiently and accurately reduces. Louder noise bursts can be more disruptive. Noise is more likely to reduce the accuracy of the work than reduce the total quantity of work done. Complex tasks are more likely to be impaired.

Hearing loss	Prolonged exposure to noise levels above 85 dBA can damage inner ear cells and lead to hearing loss. At first, hearing loss is usually temporary and recovery takes place over a few days. After further exposure, people may not fully recover and develop deafness. The extent of deafness depends on the degree of exposure and individual susceptibility. Even brief exposure to very high levels of 130 dBA or more can cause instant, irreversible hearing damage. Research has shown that noise is one of the leading causes of hearing loss for millions of people with impaired hearing in the United States.

Physiological changes	Noise can change a man's physiological state by speeding up pulse and respiratory rates. There is medical evidence that noise can cause heart attacks in individuals with existing cardiac injury and that continued exposure to loud noises could cause such chronic effects as hypertension or ulcers. According to medical studies, there is an increased risk to the cardiovascular system from a sound pressure level of above 65 dBA.

References	[12–15]

**Table 2:** Brief USEPA and WHO recommended sound levels for community noise.

Level	Effect	Area
**USEPA**		All outdoor areas including residential zones, farms, and other places where people spend varying amount of time and places in which quiet is the basis for use.
Leq(24) < 70 dB*	Hearing outdoor activity	
Ldn < 55 dB**	Interference and annoyance	
Leq (24) < 55 dB	Outdoor activity interference and annoyance	Outdoor areas where people spend limited amount of time such as school yards, and playgrounds.**

**WHO**		
Leq (24) = 55 dB	Serious to moderate annoyance	Outdoor living area
Leq (24) = 70 dB	Hearing impairment	Industrial, commercial shopping, and traffic areas, indoors and outdoors

References		[10–13]

*Level equivalent of sound (Leq) is the energy average noise level (usually A-weighted) integrated over some specified time. Also referred to as Equivalent sound pressure levels.

**Ldn = loudness.

**Table 3:** 1999 WHO assessment of community noise problem in greater Beirut area.

Land use	Noise standard dBA	
	Day time*	Evening time**
Commercial, administrative, or downtown	55–65	50–60
Residential/commercial centers on highways	50–60	45–55
City residential areas	45–55	40–50

*7:00 a.m. to 6:00 p.m., **6:00 p.m. to 10:00 p.m.

Lebanese ambient noise limits for intensity in different land use zones [14, 15].

**Table 4:** 2010 assessment of street noise in greater Beirut area.

Land use	Noise standard dBA		
	Morning*	Noon**	Afternoon***
Hamra-1 intersection next to Costa Coffee	65–75	65–75	60–70
Hamra-2 intersection at Red Shoe	65–75	60–70	60–70
Hamra-3 intersection Abou Taleb at Sadat	65–75	60–70	65–75
Bliss—in front of Penrose Gate	60–70	60–70	60–70

*7:00 a.m. to 9 a.m., **12 p.m. to 2 p.m., ***4p.m. to 6 p.m.

**Table 5:** Lebanese ambient noise limits for intensity in commercial areas.

Comparison	Mean average of noise standard dBA
1999 WHO-Study	55–65*
2010 IFI-Study	62-72* (12% sound dB and 400% increase in noise intensity)

*7:00 a.m. to 6:00 p.m.
